# Dominance and epistatic genetic variances for litter size in pigs using genomic models

**DOI:** 10.1186/s12711-018-0437-3

**Published:** 2018-12-22

**Authors:** Zulma G. Vitezica, Antonio Reverter, William Herring, Andres Legarra

**Affiliations:** 1 INP ENSAT, UMR 1388 GenPhySE, 31326 Castanet-Tolosan, France; 2 CSIRO Agriculture and Food, 306 Carmody Rd., St Lucia, QLD 4067 Australia; 3 PIC North America, Hendersonville, TN 37075 USA; 4 INRA, UMR 1388 GenPhySE, 31326 Castanet-Tolosan, France

## Abstract

**Background:**

Epistatic genomic relationship matrices for interactions of any-order can be constructed using the Hadamard products of orthogonal additive and dominance genomic relationship matrices and standardization based on the trace of the resulting matrices. Variance components for litter size in pigs were estimated by Bayesian methods for five nested models with additive, dominance, and pairwise epistatic effects in a pig dataset, and including genomic inbreeding as a covariate.

**Results:**

Estimates of additive and non-additive (dominance and epistatic) variance components were obtained for litter size. The variance component estimates were empirically orthogonal, i.e. they did not change when fitting increasingly complex models. Most of the genetic variance was captured by non-epistatic effects, as expected. In the full model, estimates of dominance and total epistatic variances (additive-by-additive plus additive-by-dominance plus dominance-by-dominance), expressed as a proportion of the total phenotypic variance, were equal to 0.02 and 0.04, respectively. The estimate of broad-sense heritability for litter size (0.15) was almost twice that of the narrow-sense heritability (0.09). Ignoring inbreeding depression yielded upward biased estimates of dominance variance, while estimates of epistatic variances were only slightly affected.

**Conclusions:**

Epistatic variance components can be easily computed using genomic relationship matrices. Correct orthogonal definition of the relationship matrices resulted in orthogonal partition of genetic variance into additive, dominance, and epistatic components, but obtaining accurate variance component estimates remains an issue. Genomic models that include non-additive effects must also consider inbreeding depression in order to avoid upward bias of estimates of dominance variance. Inclusion of epistasis did not improve the accuracy of prediction of breeding values.

## Background

Genomics provides tools to understand the effects of genes and their interactions and new approaches for genetic improvement [1]. In quantitative genetics, partitioning genetic variance for a trait into statistical components due to additivity, dominance, and epistasis is useful for prediction and selection, even if it does not reflect the biological (or functional) effect of the underlying genes [2]. Additive, dominance and epistatic genetic variances of quantitative traits are required to estimate breeding values and for making optimal selection decisions.

Within-breed non-additive effects, and in particular epistasis, are often ignored in genetic improvement programs. However, the total genetic value of an animal is a function of both additive and non-additive effects and, taken together, these effects could result in better predictors of future phenotypes [3] and inform mate allocation. Indeed, assortative mating can improve the performance of livestock when dominance [4] and/or epistasis are/is present [5]. Evidence of non-additive variance in commercially important traits (e.g. for body depth in fish, [6]) has opened opportunities for specialized breeding schemes. To take the effects of dominance and/or epistasis into account, it is necessary to refine the estimation of non-additive variance components from phenotypic data.

The additive genetic effect is the part of an individual’s total genetic effect that is transmissible across generations from parents to offspring. In contrast, non-additive genetic effects (dominance and epistasis) can be seen as the residual genetic effect after fitting additive substitution effects. Although most of the genetic variance is additive [1, 7] and captures most of the functional epistatic action of genes, the epistatic variance should not be neglected. Knowing its magnitude in real data and exploring the predictive ability of a model that accounts for epistatic effects are of great relevance. It is also of interest to know how much gene-by-gene (G × G) interaction exists (e.g. to determine the effect of the same allele in different breeds) or to account for the contribution of epistasis to the creation of “new” additive variance over time [8].

Several authors have proposed the inclusion of dominance and epistatic effects in genetic evaluation using high-density marker genotypes (genomic evaluation) [5, 9–12]. Most epistatic models consider only additive-by-additive epistatic interactions (e.g. [9, 12]), although dominance-by-dominance and dominance-by-additive interactions may play a major role in heterosis and in inbreeding and outbreeding depression (see [13] p. 223). Recently, Vitezica et al. [14] proposed a flexible and general approach to construct “genomic” relationship matrices for populations that are or are not in Hardy–Weinberg equilibrium (HWE), e.g. F1 crosses. They proved that epistatic genomic relationship matrices for two or higher order interactions can be constructed using Hadamard products of additive and dominance genomic orthogonal relationships, regardless of the existence of HWE. They also pointed out that, nevertheless, standardization of genomic relationship matrices based on the trace of the relationship matrices is needed. However to date, models that use these relationship matrices have not been applied to real data.

Xiang et al. [15] proved analytically that, in the presence of directional dominance, inclusion of genomic inbreeding as a covariate is necessary to obtain correct estimates of dominance variance. Genomic inbreeding of an individual was shown to be correctly defined as the proportion of genotyped single nucleotide polymorphisms (SNPs) at which the individual is homozygous [16]. This was confirmed in real data by Xiang et al. [15] and Aliloo et al. [4]. The effect of fitting or not fitting genomic inbreeding on estimates of epistatic variance components is unknown.

In current pig production systems, the number of pigs weaned is a key factor to increase productivity, and litter size (e.g., total number of piglets born per litter) is one of the most important traits under selection, in maternal line breeding programs [17]. Although litter size in pigs has a low narrow-sense heritability [17–19], non-additive variance could still be abundant and should be ascertained. This is particularly important because the estimation of non-additive effects (dominance and epistasis) for litter size is of increasing interest since it can be used to define mate allocation strategies between selection candidates for developing new crossbreeding or even purebred breeding schemes.

The objectives of this work were to estimate additive and non-additive (dominance and epistasis) variance components for litter size for a real pig population, by considering or not inbreeding depression and to determine whether the accuracy of prediction of breeding values and total genetic values increases with the inclusion of dominance and epistatic effects.

## Methods

### Phenotypic and genomic data

Data for this study were provided by Genus plc (Hendersonville, TN, USA). Animal Care and Use Committee approval was not necessary for this study because the data were obtained from an existing database. Data on litter size (total number of piglets born per litter) were from a pig pure line. The average litter size was equal to 12.7 ± 3.1 and 13,369 records were available for 3619 sows. Genotypes for all sows were generated using the Illumina PorcineSNP60 BeadChip (Illumina, San Diego, CA). After quality control using default parameters by preGSf90 [20] (HWE, minor allele frequency, SNP call rate and animal call rate), 38,779 autosomal SNPs remained and were used to build genomic relationship matrices.

### Genomic evaluation models

Phenotypes were analyzed using a genomic best linear unbiased prediction (GBLUP) (mixed) model. Parity number and the combined effect farm-year-month of farrowing were included as fixed effects. The model also included a random permanent environmental effect for each sow. The linear model including additive, dominant and interaction terms can be written as:

$${\mathbf{y}} = {\mathbf{X\varvec{\upbeta }}} + {\mathbf{f}}b + {\mathbf{Zg}}_{A} + {\mathbf{Zg}}_{D} + {\mathbf{Z}}\mathop \sum \limits_{i = A,D} \mathop \sum \limits_{{\begin{array}{*{20}c} {j = A,D} \\ {i \ge j} \\ \end{array} }} {\mathbf{g}}_{ij} + {\mathbf{Zpe}} + {\mathbf{e}},$$

where ${\mathbf{y}}$ is the vector of phenotypic records, ${\varvec{\upbeta}}$ is the fixed effect vector, ${\mathbf{f}}b$ models the inbreeding depression, where ${\mathbf{f}}$ is the vector of genomic inbreeding coefficients based on the proportion of homozygous SNPs and $b$ is the inbreeding depression parameter per unit of inbreeding, ${\mathbf{g}}_{A}$ is a vector of breeding values of the sows, ${\mathbf{g}}_{D}$ is a vector of dominance deviations, ${\mathbf{g}}_{ij}$ is a vector of epistatic genetic values, ${\mathbf{pe}}$ is the permanent environmental effect vector $\left( {Var\left( {{\mathbf{pe}}} \right) = {\mathbf{I}}\sigma_{pe}^{2} } \right)$, ${\mathbf{e}}$ is a residual vector $\left( {Var\left( {\mathbf{e}} \right) = {\mathbf{I}}\sigma_{e}^{2} } \right)$, and ${\mathbf{X}}$ and ${\mathbf{Z}}$ are design matrices relating records to fixed effects and genetic and permanent environmental effects, respectively. The epistatic genetic effects can be partitioned into additive-by-additive $({\mathbf{g}}_{AA} )$, additive-by-dominance $({\mathbf{g}}_{AD} )$, and dominance-by-dominance $({\mathbf{g}}_{DD} )$ effects. Higher order epistasis interactions were not considered as they were deemed to be negligible [21] or too difficult to estimate. Covariance matrices of genetic effects were as follows:

$$Var\left( {{\mathbf{g}}_{A} } \right) = {\mathbf{G}}_{A} \sigma_{A}^{2} ,\quad Var\left( {{\mathbf{g}}_{D} } \right) = {\mathbf{G}}_{D} \sigma_{D}^{2} ,\quad Var\left( {{\mathbf{g}}_{AA} } \right) = {\mathbf{G}}_{AA} \sigma_{AA}^{2} ,\quad Var\left( {{\mathbf{g}}_{AD} } \right) = {\mathbf{G}}_{AD} \sigma_{AD}^{2} ,\quad Var\left( {{\mathbf{g}}_{DD} } \right) = {\mathbf{G}}_{DD} \sigma_{DD}^{2}$$

where the covariance matrices ${\mathbf{G}}_{A}$ and ${\mathbf{G}}_{D}$ for additive and dominance effects, which are involved in the construction of covariance matrices ${\mathbf{G}}_{AA}$, ${\mathbf{G}}_{AD}$ and ${\mathbf{G}}_{DD}$ for epistatic effects, were constructed assuming HWE. The additive relationship matrix was calculated as in VanRaden [22]: ${\mathbf{G}}_{A} = \frac{{{\mathbf{MM}}^{{\mathbf{\prime }}} }}{{2\sum p_{i} q_{i} }}$, where matrix ${\mathbf{M}}$ has elements that are equal to $\left( {2 - 2p_{i} } \right), \left( {1 - 2p_{i} } \right), - 2p_{i}$ for genotypes $A_{1} A_{1}$, $A_{1} A_{2}$ and $A_{2} A_{2}$, respectively, where $p_{i}$ is the frequency of allele $A_{1}$ at SNP $i$. The dominance relationship matrix was computed as ${\mathbf{G}}_{D} = \frac{{{\mathbf{WW}}^{{\mathbf{\prime }}} }}{{4\mathop \sum \nolimits_{\varvec{i}} p_{i}^{2} q_{i}^{2} }}$ where ${\mathbf{W}}$ has elements equal to $- 2q_{i}^{2} , 2p_{i} q_{i} , - 2p_{i}^{2}$ for genotypes $A_{1} A_{1}$, $A_{1} A_{2}$ and $A_{2} A_{2}$, respectively [10]. Covariance matrices for epistatic effects were computed using the Hadamard products and traces as ${\mathbf{G}}_{AA} = \frac{{{\mathbf{G}}_{A} \odot {\mathbf{G}}_{A} }}{{tr\left( {{\mathbf{G}}_{A} \odot {\mathbf{G}}_{A} } \right)/n}}$, ${\mathbf{G}}_{AD} = \frac{{{\mathbf{G}}_{A} \odot {\mathbf{G}}_{D} }}{{tr\left( {{\mathbf{G}}_{A} \odot {\mathbf{G}}_{D} } \right)/n}}$, and ${\mathbf{G}}_{DD}=\frac{{{\mathbf{G}}_{D} \odot {\mathbf{G}}_{D} }}{{tr\left( {{\mathbf{G}}_{D} \odot {\mathbf{G}}_{D} } \right)/n}}$ [14].

In these analyses, we implicitly assume that allele substitution effects of quantitative trait loci (QTL) are distributed as ${\varvec{\upalpha}}{ \sim }N\left( {0,{\mathbf{I}}\sigma_{\alpha }^{2} } \right)$, dominance effects as ${\mathbf{d}}{ \sim }N\left( {0,{\mathbf{I}}\sigma_{d}^{2} } \right)$, additive-by-additive epistatic effects as $\left( {{\varvec{\upalpha \upalpha }}} \right){ \sim }N\left( {0,{\mathbf{I}}\sigma_{{\left( {\alpha \alpha } \right)}}^{2} } \right)$, and similarly for additive-by-dominant $\left( {{\mathbf{\alpha d}}} \right)$ and dominant-by-dominant $\left( {{\mathbf{dd}}} \right)$ effects. Note that under the assumption of HWE, the GBLUP “breeding” model in Vitezica et al. [10] that accounts for additive and dominance effects is a particular case of the model to analyze epistasis in Vitezica et al. [14], which in turn is an extension of the NOIA (natural orthogonal interactions) QTL analysis model by Alvarez-Castro and Carlborg [23]. Note that a “statistical” or “breeding” model implies that the covariance between breeding values and dominance deviations is zero [24]. The NOIA model is orthogonal, which means that genetic effects are defined in such a way that the addition of other genetic effects (e.g. dominance) to a model does not change the definitions of genetic effects (e.g. additive) that were already included in the model [23]. For instance, the additive effect is always the regression of genotypic value on gene content; the dominance effect is a regression of the remainder on a measure of heterozygosity or identity at the genotype, and so on. The NOIA model of Alvarez-Castro and Carlborg [23] achieves orthogonality automatically due to its construction of the incidence matrices ${\mathbf{M}}$ and ${\mathbf{W}}$ and their Kronecker products; Vitezica et al. [14] proved that, for analyses at the individual level, these Kronecker products can be reformulated as Hadamard products.

Statistically, orthogonality means that inclusion of new terms in the model does not change estimates of already included effects in an infinitely large population. For instance, in practice, going from an additive to an additive plus dominant model should not change the estimates of additive variance components much. The advantage of using orthogonality in genetics and breeding is that it is the only way to carry out the estimation of breeding values (additive “statistical” effects) in an unambiguous manner i.e. such that they do not depend on other genetic terms that are fitted in the model.

Variance components were estimated for five nested models that added, in succession, additive effects ($A$), dominance effects ($A + D$), additive-by-additive genetic effects ($A + D + AA$), additive-by-dominance genetic effects ($A + D + AA + AD$), and dominance-by-dominance genetic effects ($A + D + AA + AD + DD$). Genetic variances ($\sigma_{A}^{2}$, $\sigma_{D}^{2}$, $\sigma_{AA}^{2}$, $\sigma_{AD}^{2}$ and $\sigma_{DD}^{2}$) were estimated by Bayesian methods using Gibbs sampling in the software gibbs2f90 [25], available at http://nce.ads.uga.edu/wiki/. In total, 200,000 iterations were run, discarding the first 10,000 and keeping every 100th sample. Convergence was checked by visual inspection of trace plots for the chains. Post-Gibbs analysis included estimation of the effective sample size and the deviance information criteria (DIC) as a goodness-of-fit criterion [26].

### Predictive ability

GBLUP was used to obtain estimated breeding values (EBV) by fixing the variance components that were estimated. Predictive ability of total genetic values for lowly heritable traits is difficult to ascertain, thus for the comparison of models, we proceeded as follows. EBV $\left( {{\hat{\mathbf{g}}}_{A}^{w} } \right)$ were obtained from the “whole” dataset (13,369 records from 3619 sows), which included litters from 2000 to 2014. EBV $\left( {{\hat{\mathbf{g}}}_{A}^{p} } \right)$ were also computed for a “partial” dataset that included only litters prior to 2010 (10,002 records from 2440 sows). No sow farrowed both before and after 2010 (such sows were excluded from the data set). The three models: $A$, $A + D$, and $A + D + AA + AD + DD$ were used to estimate ${\hat{\mathbf{g}}}_{A}^{w}$ and ${\hat{\mathbf{g}}}_{A}^{p}$. The models were compared using the following three statistics of cross-validation based on method R approaches [27, 28] to detect bias in subsequent genetic evaluations, and as more recently suggested by [29]:

$$\begin{aligned} b_{0} & = \left( {1^{{\prime }} {\hat{\mathbf{g}}}_{A}^{w} - 1^{{\prime }} {\hat{\mathbf{g}}}_{A}^{p} } \right) /n, \\ b_{1} & = \frac{{Cov \left( {{\hat{\mathbf{g}}}_{A}^{w} ,{\hat{\mathbf{g}}}_{A}^{p} } \right)}}{{Var\left( {{\hat{\mathbf{g}}}_{A}^{p} } \right)}} , \\ \rho & = \frac{{Cov \left( {{\hat{\mathbf{g}}}_{A}^{w} ,{\hat{\mathbf{g}}}_{A}^{p} } \right)}}{{\sqrt {Var\left( {{\hat{\mathbf{g}}}_{A}^{w} } \right)Var\left( {{\hat{\mathbf{g}}}_{A}^{p} } \right)} }}, \\ \end{aligned}$$

where $b_{0} = 0$ is referred to as the bias and should be equal to 0 under unbiasedness; $b_{1}$ is a measure of dispersion of EBV, obtained as the slope of the regression of EBV obtained with the whole data on EBV estimated with the partial data, and should be 1; and $\rho$ measures the relative increase of accuracies of EBV from partial data to whole data and was computed as the correlation of partial data on whole (among two models with different values of $\rho$, the model with the highest $\rho$ was chosen). EBV of only “new” sows that farrowed after 2010 were included in the statistics. These sows have no own or progeny phenotypic information in the partial data and thus, they can be seen as selection candidates at birth.

For comparison purposes, we also investigated the predicted ability of phenotypes of “new” sows for the three models as the correlation $cor\left( {y^{*} ,\hat{y}} \right)$ [30] where $y^{*}$ is the corrected phenotypic observation obtained from the “whole” dataset $\left( {y^{*} = y - X\hat{\beta } - f\hat{b}} \right)$, and $\hat{y}$ is the predicted corrected observation from the “partial” dataset, which is equal to the sum of the estimated genetic values $\left( {\hat{g}} \right)$ and the estimated permanent environmental effect $\left( {\widehat{pe}} \right)$. The estimated genetic value included the estimated additive genetic effects ($A$ model), the sum of estimated additive and dominant genetic effects ($\hat{g} = \widehat{{g_{A} }} + \widehat{{g_{D} }})$ in the $A + D$ model, and the sum of all genetic effects $(\hat{g} = \widehat{{g_{A} }} + \widehat{{g_{D} }} + \widehat{{g_{AA} }} + \widehat{{g_{AD} }} + \widehat{{g_{DD} }}$), in the $A + D + AA + AD + DD$ model. The regression coefficients of $y^{*}$ on $\hat{y}$ were also estimated.

## Results and discussion

**Fig. 1:**
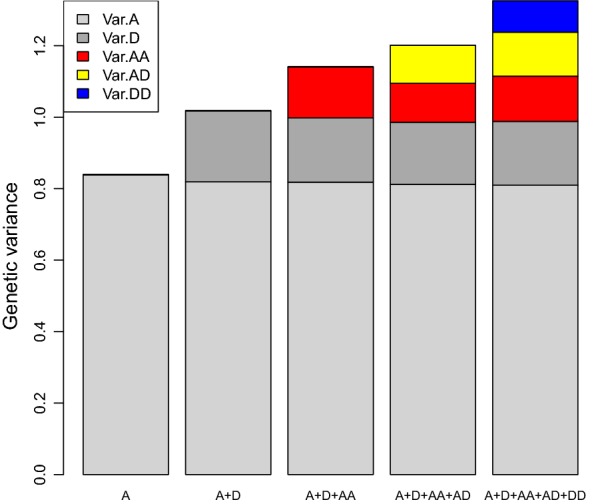
Estimates of additive (Var.A), dominance (Var.D), additive-by-additive (Var.AA), additive-by-dominance (Var.AD), and dominance-by-dominance (Var.DD) genetic variances for five models that included genomic inbreeding and successively added additive effects ($A$), dominance effects ($A + D$), additive-by-additive effects ($A + D + AA$), additive-by-dominance effects ($A + D + AA + AD$), and dominance-by-dominance effects ($A + D + AA + AD + DD$)

Estimates of additive and dominance genetic variances for litter size ranged from 0.81 ± 0.12 to 0.84 ± 0.12, and from 0.17 ± 0.11 to 0.20 ± 0.11, respectively for all models ($A$, $A + D$, $A + D + AA$, $A + D + AA + AD$ and $A + D + AA + AD + DD$). Variance component estimates did not differ among the models (Fig. 1), which empirically illustrates the orthogonality in the partition of the total genetic variance, a property that holds under HWE (which holds in this dataset) and under linkage equilibrium (which holds approximately). Under orthogonality, allele substitution effects contribute to the additive variance, dominance deviations contribute to the dominance variance, etc. and there is no covariance between the genetic effects. If the model used is not orthogonal (e.g. “genotypic” model in Su et al. [9]), estimates of additive genetic variance may be biased downward when the model is expanded from additive to include dominance and epistatic effects (e.g. Su et al. [9] and Muñoz et al. [31]), and many others). Not using orthogonal models leads to views that are too optimistic on the role of dominance on breeding.

Estimates of epistatic variance ranged from 0.11 ± 0.11 to 0.14 ± 0.12 for the additive-by-additive component, from 0.11 ± 0.09 to 0.12 ± 0.09 for the additive-by-dominant component, and were equal to 0.09 ± 0.09 for the dominant-by-dominant component. As explained in many previous papers [1, 7], the magnitude of the epistatic variances is trivial compared to that of the additive variance. However, non-orthogonal models can result in exaggerated estimates of epistatic variances (e.g. Su et al. [9] and Muñoz et al. [31]). Estimates of epistatic variances had a large standard error in all models ($A + D + AA$, $A + D + AA + AD$ and $A + D + AA + AD + AA$), which illustrates the difficulties in obtaining good estimates of epistatic variances also from genomic information, even when there are only two-way interactions.

Estimates of narrow-sense heritability for the total number of piglets born per litter were similar between models (Table 1), close to 0.09, and consistent with estimates reported by Varona et al. [32], Nielsen et al. [17] and Guo et al. [18]. Dominance variance expressed as a proportion of phenotypic variance, $d^{2}$, was about 0.02, as in Misztal et al. [33]. The total epistatic variance (additive-by-additive plus additive-by-dominance plus dominance-by-dominance) expressed as a proportion of phenotypic variance, $i^{2}$, was 0.04. The latter estimates need to be considered with caution due to the low precision of the estimates of epistatic effects. The estimated broad-sense heritability for litter size $\left( {h^{2} + d^{2} + i^{2} } \right)$ was 0.15, i.e. almost twice the narrow-sense heritability.

**Table 1:** Estimates (and posterior standard deviation) of narrow sense heritability and of variance components for models that included genomic inbreeding and successively added additive effects ($A$), dominance effects ($A + D$), additive-by-additive effects ($A + D + AA$), additive-by-dominance effects ($A + D + AA + AD$), and dominance-by-dominance effects ($A + D + AA + AD + DD$)

Model	$h^{2}$	$d^{2}$*	$i^{2}$**	$\sigma_{pe}^{2}$	$\sigma_{e}^{2}$
$A$	0.095 (0.013)			0.931 (0.110)	7.049 (0.116)
$A + D$	0.093 (0.013)	0.022 (0.013)		0.755 (0.143)	7.053 (0.117)
$A + D + AA$	0.093 (0.013)	0.020 (0.010)	0.016 (0.015)	0.633 (0.178)	7.052 (0.117)
$A + D + AA + AD$	0.092 (0.013)	0.020 (0.011)	0.024 (0.014)	0.572 (0.179)	7.051 (0.118)
$A + D + AA + AD + DD$	0.092 (0.013)	0.019 (0.012)	0.038 (0.017)	0.450 (0.184)	7.054 (0.117)

* $d^{2} = \sigma_{D}^{2} /\sigma_{P}^{2}$, ** $i^{2} = \sigma_{AA}^{2} /\sigma_{P}^{2}$ for the $A + D + AA$ model, $i^{2} = \left( {\sigma_{AA}^{2} + \sigma_{AD}^{2} } \right) /\sigma_{P}^{2}$ for the $A + D + AA + AD$ model, and $i^{2} = \left( {\sigma_{AA}^{2} + \sigma_{AD}^{2} + \sigma_{DD}^{2} } \right) /\sigma_{P}^{2}$ for the $A + D + AA + AD + DD$ model

**Fig. 2:**
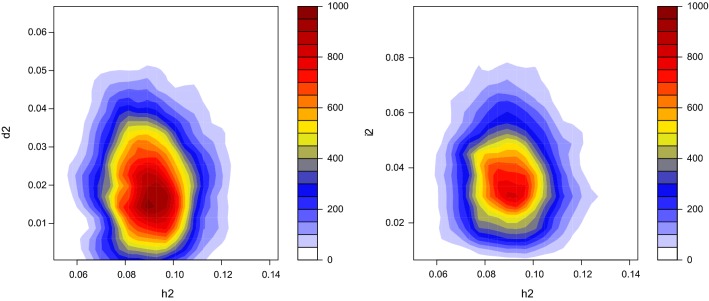
Bivariate density plot of the posterior distribution (1900 samples) of narrow sense heritability $\left( {h^{2} } \right)$, dominance variance as a proportion of phenotypic variance $\left( {d^{2} = \sigma_{D}^{2} /\sigma_{P}^{2} } \right)$, and total epistatic variance as a proportion of phenotypic variance $\left( {i^{2} = \left( {\sigma_{AA}^{2} + \sigma_{AD}^{2} + \sigma_{DD}^{2} } \right)/\sigma_{P}^{2} } \right)$ for the $A + D + AA + AD + DD$ model

The joint posterior distribution of heritability and $d^{2}$ or $i^{2}$ (Fig. 2) shows that there is no dependency between variance component estimates and thus, the partition of additive and non-additive effects was empirically orthogonal.

Inclusion of non-additive (dominance and epistasis) effects in the model did not have a large effect on estimates of residual variance, but they reduced the permanent environmental variance (Table 1), which shows that non-additive genetic effects are captured by the permanent environmental effects if they are not included explicitly in the model. Similar results were observed by Aliloo et al. [4] when dominance was included in an additive model.

Only second-order epistatic effects (e.g. additive-by-additive) were included in this study. Although it is tempting to fit high-order epistasis terms given the relative ease of computing the Hadamar products of relationship matrices, caution is needed. First, the products ${\mathbf{G}} \odot {\mathbf{G}} \odot {\mathbf{G}} \ldots$ quickly tend to the identity matrix, in which case there is no hope of distinguishing genetic components from residual effects. Second, partitioning genetic variance into additive and non-additive statistical components does not indicate the importance of additive versus non-additive gene actions [2].

**Fig. 3:**
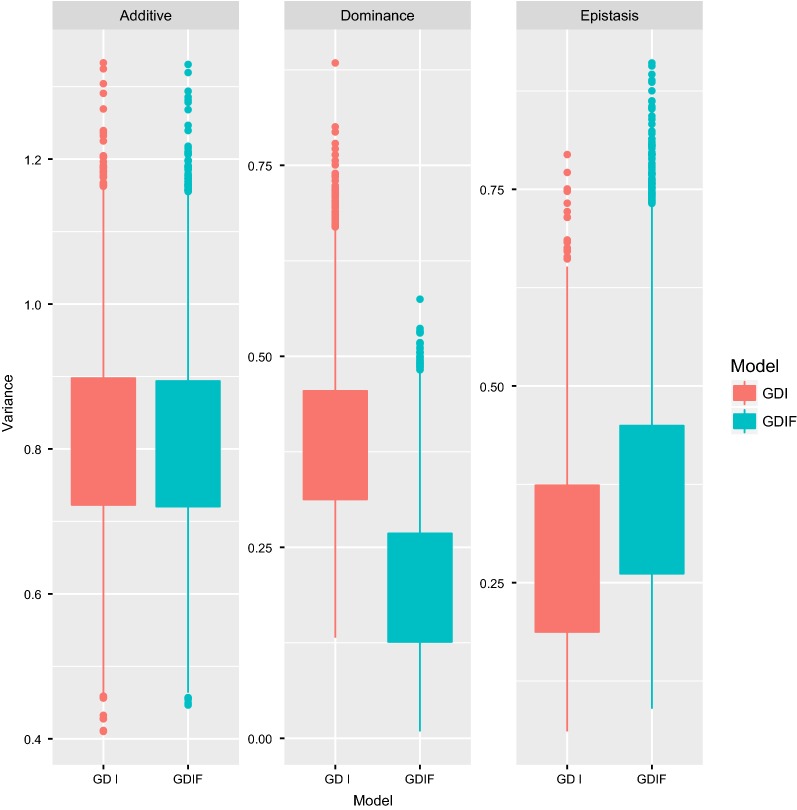
Estimates (boxplots of posterior distributions) of additive, dominance, and epistatic genetic variances for the $A + D + AA + AD + DD$ model, with (GDIF) or without (GDI) including genomic inbreeding. The $A + D + AA + AD + DD$ model involves additive, dominance, additive-by-additive, additive-by-dominance, and dominance-by-dominance effects

The estimate of inbreeding depression, based on the inbreeding coefficient calculated as the proportion of homozygosity, was equal to − 12.33 ± 2.29 piglets born per litter for this population. Inbreeding depression expressed as a change in phenotypic mean per 10% increase in inbreeding was equal to − 1.23 piglets born. This result shows the importance of inbreeding depression in fitness traits such as total number of piglets born. Estimates of non-additive variance components when inbreeding depression is not fitted in the model are rarely reported but, in our analyses, yielded an upward biased estimate of the dominance variance (i.e. GDI model in Fig. 3). The estimate of dominance variance increased from 0.18 in the GDIF model (including inbreeding) to 0.38 in the GDI model. The estimate of epistatic variance was slightly affected by inclusion of inbreeding depression in the model. Estimates of additive variance were not affected. Similar results for dominance variance were obtained by Xiang et al. [15] and Aliloo et al. [4]. To take directional dominance into account, inbreeding depression must be included in genetic evaluation models, which has long been known for pedigree analysis (e.g. DeBoer and Hoeschele [34]).

The global fit of the models was studied using DIC. Models with smaller DIC exhibit a better fit after accounting for model complexity. Differences in DIC between models of less than 7 units are considered as irrelevant [35]. The values of DIC were 68,365, 68,370, 68,367, 68,370 and 68,375 for the $A$, $A + D$, $A + D + AA$, $A + D + AA + AD$ and $A + D + AA + AD + AA$ models, respectively, i.e. they were similar across models, and models that included dominance and epistasis do not appear to fit the data better than the simplest model.

The predictive ability of the models for selection candidates was assessed using two approaches: the three statistics based on method R and by conventional cross-validation. The method R statistics of bias $\left( {b_{0} } \right)$, slope $\left( {b_{1} } \right)$, and correlation $\left( \rho \right)$ were based on comparison of EBV obtained from the whole data (up to 2014) and model $A$ with EBV obtained from the partial data and models $A$, $A + D$ and $A + D + AA + AD + AA$. Similar results (not shown here) were obtained when using the $A + D$ and $A + D + AA + AD + DD$ models in the whole dataset. For the correct models, $b_{0} = 0$ and $b_{1} = 1$ are expected. The estimate of $b_{0}$ was equal to $0.019\sigma_{A}$ across the models and the estimate of $b_{1}$ was equal to 1.01, 1.04 and 1.09 in models $A$, $A + D$ and $A + D + AA + AD + DD$, respectively. These estimates near zero for $b_{0}$ and near to 1 for $b_{1}$, suggest that the model is empirically unbiased. The statistic $\rho$ (accuracy), measured as the correlation between the EBV of selection candidates based on “whole” and “partial” data, was around 0.73 for all models.

Based on conventional cross-validation (Table 2), models $A$, $A + D$ and $A + D + AA + AD + DD$ showed similar predictive ability of phenotypes. Estimates of the regression coefficient of $y^{*}$ on $\hat{y}$ were close to 1 for all three models, which all achieved very similar levels of inflation of total genetic values. No differences in the accuracy of predicting future records for young animals were observed between the three models.

**Table 2:** Predictive ability of phenotype for different models

Model	G^*^	GD^**^	GDI^***^
$cor\left( {y^{*} ,\hat{y}} \right)$ ^a^	0.135	0.136	0.136
Regression coefficient^b^	0.896	0.922	0.978

^a^Predictive ability $\left( {cor\left( {y^{*} ,\hat{y}} \right)} \right)$ is measured as the correlation between the corrected phenotypic observation $\left( {y^{*} } \right)$ and the predicted corrected observation $\left( {\hat{y}} \right)$

^b^Regression coefficients of $y^{*}$ on $\hat{y}$

* G is for the additive model, **GD is for the $A + D$ model, *** GDI is for the $A + D + AA + AD + DD$ model

## Conclusions

Using orthogonal relationship matrices, empirically orthogonal estimates of additive, dominance and epistatic variances were obtained for litter size in a pig dataset. The broad-sense heritability for litter size was almost twice the narrow-sense heritability. Genomic models that include non-additive effects must consider simultaneously inbreeding depression based on inbreeding in order to obtain unbiased estimates of variance components. Inclusion of epistasis or dominance did not improve the accuracy of prediction of breeding or genotypic values.
